# Complex Mixtures and Multiple Stressors: Evaluating Combined Chemical Exposures and Cumulative Toxicity

**DOI:** 10.3390/toxics11060487

**Published:** 2023-05-26

**Authors:** Christopher D. Kassotis, Allison L. Phillips

**Affiliations:** 1 Institute of Environmental Health Sciences, Department of Pharmacology, Wayne State University, Detroit, MI 48202, USA; 2 Center for Public Health and Environmental Assessment, U.S. Environmental Protection Agency, Corvallis, OR 97333, USA

The problem of chemical mixtures in the environment encompasses biological, analytical, logistical, and regulatory challenges, among others. Components of contaminant mixtures can produce additive effects and, although less frequently reported, can interact to produce effects that are greater than or less than those predicted by additivity (synergistic or antagonistic effects, respectively) [1,2,3]. As has been demonstrated, combinations of low- or “no”-activity chemicals can act additively or synergistically to elicit significant, measurable effects and/or can modulate the effects of endogenous hormone activity [1,2]. Biomonitoring studies continue to report routine human exposure to hundreds or thousands of chemicals through the integration of exposomics and metabolomics [4,5,6,7], underscoring the incredible complexity involved in understanding real-world chemical mixture exposures. Such complexity presents practical barriers, as research efforts cannot possibly examine every contaminant mixture, given the unique chemical exposure profile experienced by each individual person. With an estimated >350,000 chemicals and mixtures registered on the global market, the number of unique mixture combinations that could potentially be tested is staggering [8]. Research tends to overlook whole mixtures in toxicological testing, with >80% of mixture studies focusing on small, technically simple mixtures of two or three similar components [3]. These realities have driven the prevalence of and reliance on component-based approaches in the field of mixture risk assessment. While the evaluation of defined chemical mixtures increases our understanding of chemical interactions and generates potentially useful data for mixture assessments, these simple mixtures often lack environmental relevance. To robustly address human and ecological exposure to environmental mixtures such as floodwaters, wildfire smoke, or house dust, and ultimately reduce the uncertainty associated with mixture risk and hazard estimates, we must advance the state of the science on mixtures research and risk assessment.

*Critical Reviews of Mixture Research.* This Special Issue brings together cutting-edge publications that have expanded our understanding of complex contaminant mixtures and health outcomes. Several efforts were undertaken to more comprehensively evaluate the existing literature relating to different facets of mixture effects on human and environmental health. Yim et al., 2022 performed a scoping review to evaluate the potential impact(s) of metal mixtures on cardiovascular disease risk factors and outcomes among non-occupationally exposed populations [9]. This analysis revealed four focus areas, including blood pressure and/or hypertension, pre-eclampsia, dyslipidemia and/or serum lipids, and stroke incidence and/or coronary heart disease [9]. Their analysis of the literature suggested possible cardiotoxicity from exposures to metal mixtures (often distinct from constituent single contaminants), supporting the necessity for further evaluation of complex contaminant mixtures that better reflect environmental relevance [3]. A contribution from Trevisan and Ranasinghe et al., 2022 discusses the current state of knowledge on nanoplastics in the aquatic environment, detailing effects on aquatic species and the interplay of these particles with environmental contaminants and other stressors, such as temperature, salinity, pH, organic matter, and food availability [10]. Nanoplastics experiments face challenges similar to those encountered by studies of organic pollutant mixtures, where the majority of research has evaluated defined mixtures of similar sizes under controlled conditions that may not be representative of actual environmental occurrence. Another key limitation in this area of research is knowledge of how nanoplastics interact with other environmental contaminants. These dynamics are still uncertain and may result in enrichment of the availability of specific subsets of contaminants in the environment [10]. Lastly, Fletcher et al., 2022 presented a comprehensive review of phthalate mixtures on ovarian folliculogenesis and steroidogenesis [11]. This review presents a deep dive into a specific set of common co-occurring contaminants with well-described effects on female reproductive health. Similar themes were observed in this review, with authors articulating a need for more environmentally realistic research on common co-occurring phthalates at proportions that reflect human exposures (e.g., based on proportions and including concentrations reported in human cohort studies [12,13]), as well as a better understanding of mechanistic determinations of causal chemicals underlying complex mixture-mediated effects on female reproduction [11]. However, there are some inherent challenges in attempts such as these, where exposures may range widely depending on specific populations (e.g., demographic characteristics, physiological conditions, etc.) [14]; as such, defining a realistic environmental mixture may only apply to certain parts of the population and relies on rigorous biomonitoring efforts for the pollutants of interest.

*In vitro Contributions and New Approach Methodologies.* New approach methodologies (NAMs) are technologies and approaches that do not use test animals and can provide information useful in risk assessments. NAMs offer the potential to improve mixture assessment and reduce residual uncertainty by lowering the cost of toxicity testing, incorporating human-based models of disease, facilitating mechanistic evaluation, and making mixture data more rapidly available to decision makers [15]. Several publications in this Special Issue leveraged NAMs, ranging from in vitro bioassays to cheminformatic approaches and in silico predictive modeling, to better understand mixture-related effects. Perez et al., 2022 investigated the combined effects of bisphenol A (BPA) and several of its substitutes (Bisphenol F and S [BPF and BPS]) in an in vitro model of obesogenic activity [16]. An equimolar mixture of bisphenols altered intracellular lipid accumulation and mRNA expression of genes and proteins related to adipogenesis in a manner distinct from individual bisphenols, highlighting the need for consideration of the potential cumulative effects of similar chemicals. Using the U.S. Environmental Protection Agency’s Chemical and Products Database (CPDat) and the ToxCast/Tox 21 databases, Carberry et al., 2022 identified benzyl cinnamate, butyl paraben, decanoic acid, eugenol, and sodium dodecyl sulfate as chemicals that co-occur in common exposure sources and target peroxisome proliferator activated receptor gamma (PPARγ) [17]. They examined the effect of these chemicals individually and in mixtures on PPARγ expression in human liver cells. Similar to the findings of Perez et al., Carberry et al. observed that PPARγ expression was increased significantly as a result of mixture exposure. The authors presented a unique approach to informing study design by combining cheminformatic approaches with in vitro bioactivity testing. In a similar vein, Ha et al., 2022 coupled biomonitoring with in vitro testing to quantify a suite of flame retardants collected by passive air samplers deployed in global megacities and tested the effect of air sample extracts on cytotoxicity and gene expression in chicken embryonic hepatocytes [18]. Weak relationships between flame retardant profiles and gene expression effects were observed, emphasizing the challenges of predicting the biological behavior of environmentally relevant and complex mixtures. The approaches used by these authors offer a path forward for generating high-throughput mechanistic data for combinations of environmental chemicals and will be paramount to filling data gaps provided by traditional toxicity testing of mixtures.

*In vivo Contributions.* While building confidence in NAM data in necessary to transition to less costly and higher-throughput toxicity testing, traditional in vivo models of toxicity remain the current gold standard for data supporting mixture risk evaluations. This Special Issue also brings together a set of publications detailing cutting-edge mixture research using both rodent and zebrafish models. Merrill et al., 2021 utilized a mixture of four endocrine-disrupting chemicals (atrazine, bisphenol A, perfluorooctanoic acid, and 2,3,7,8-tetrachlorodibenzo-p-dioxin) that were likely to co-occur in a pregnancy exposure model in mice and compared exposure-induced effects between both pregnant and non-pregnant dams, assessing the impact of pregnancy as a potential non-chemical stressor [19]. The mixture induced metabolic health effects (e.g., glucose intolerance, increased weight, visceral adiposity, and serum lipids) in the exposed dams, but only in those exposed during pregnancy, supporting the concept of a complex stressor (chemical exposure during a critical physiological window) potentially producing more significant effects than traditional chemical(s) exposure [19]. Two other contributions used multiple stressor mixture exposure study designs. Gillera et al., 2022 combined complex environmental stressors, coupling both a flame retardant mixture exposure (Firemaster 550) [20] with the early life social stressor of paternal absence in the prairie vole [21]. Following individual and/or combined stressor exposures, adult offspring were subjected to a battery of tests to evaluate prosocial behaviors. The flame retardant mixture exposure resulted in increased anxiety and partner preference in females as well as decreased partner preference in males, whereas the paternal deprivation caused increases in anxiety, decreases in sociability, and impairments of pair bonding in both sexes [21]. Interestingly, the combination of these chemical and social stressors increased some prosocial behaviors while inhibiting others, supporting the idea of unanticipated complex mixture effects on behavioral outcomes. Gore et al., 2022 also took a novel approach, selecting a mixture of bisphenols, phthalates, vinclozolin, and perfluorinated, polybrominated, and polychlorinated compounds, each with individual evidence of neurodevelopmental impacts, and designed the mixture to represent realistic environmental exposures (e.g., chemicals detected in the majority of humans and at doses below no observed adverse effect levels) [22]. Following gestational exposure, a “second-hit” stress challenge was employed to assess chemical-induced perturbations to stress sensitivity. The chemical mixture affected anxiety, social, and mate preference behaviors in females but not males, while the stress effects were primarily observed in the males [22]. Statistical interactions between the two exposures were observed for mate preference and brain gene expression, supporting the need to better understand more realistic and complex chemical and multiple stressor exposures.

Two other manuscripts assessed effects of chemical mixture exposures in the vertebrate zebrafish model. Fey et al., 2022 assessed binary mixtures of two per-/poly-fluoroalkyl substances during early embryonic development both individually and in combination, at environmentally relevant exposure concentrations [23]. The authors demonstrated that the relative potency of perfluorooctanesulfonic acid (PFOS) to 6:2 fluorotelomer sulfonic acid (6:2 FTS) for changes in swim bladder area did not exhibit constant proportionality, but instead varied as a function of the dose range. This manuscript reinforces central themes of this issue, in particular the important nuances of predicting contaminant mixture responses from component data. Lastly, Kassotis et al., 2022 performed early developmental exposures in zebrafish to technical alkylphenol polyethoxylate mixtures and examined metabolic health outcomes [24]. These technical mixtures, which are commonly used in consumer product applications, are comprised of a base alkylphenol with varying degrees of ethoxylation, resulting in complex mixtures of polyethoxylates with varying alkyl and ethoxylate chain lengths. Perhaps surprisingly, the adipogenic and the obesogenic activities increased with increasing ethoxylate chain length, then decreased with the longest chain lengths, demonstrating a non-monotonic response curve where the highest and lowest chain lengths had considerably lower effects as compared to the medium chain lengths [24], similar to effects reported previously for medium-chain-length phthalates [25,26].

*Translating Mixture Effects to Humans and Ecosystems.* Translating the findings of mixture studies to decision-making is a difficult undertaking, especially in light of the limited (and sometimes, conflicting) research available. Dose addition and response addition are two models of additivity commonly applied in risk assessment of mixtures [27]. Although there is wide support for dose addition being a health-protective model when assessing mixtures of toxicologically similar constituents [3,28,29], experimental departures from this mathematically based model have been reported [30,31]. As Fey et al., 2022 observed in their study of PFOS and 6:2 FTS in zebrafish embryos, relative potency can vary as a function of dose [23]. Instead of relying on a point estimate of relative potency (e.g., using ratios of discrete points of departure, PODs), the authors urge the use of relative potency factor estimates calculated across the full dose range when the dose–response curves of component chemicals are dissimilar. Indeed, best practices dictate that relative potency should be calculated from dose–response curves that are similarly shaped or at low response levels to reduce possible influences of high dose on the relative potency factor [27,32]. The shapes of component dose–response curves and toxicological mode of action should be interpreted jointly when considering evidence of toxicological similarity for the application of dose or response additivity models. To more accurately predict mixture behavior, data on both individual components and environmentally relevant mixtures are needed. To overcome limitations of in vivo mixture data availability, Lambert (2023) recommended leveraging existing information (including data generated in vitro and in silico) using a novel “Adverse Outcome Pathway (AOP) footprinting” approach. The AOP footprinting approach refines the mathematical basis of dose additivity, providing toxicological justification for the cumulative assessment of chemicals affecting common pathways, with data integration across multiple levels of biological organization.

Current criteria for grouping chemicals in dose additive evaluations are commonly based on chemical structure, molecular mechanisms, or apical endpoints, with evidence of structural similarity, identical molecular initiating events, toxicity in a common target organ, or shared mode of action warranting the formation of similar groups. However, important tradeoffs exist and must be considered in the regulation of chemical mixtures. If grouping criteria are set too inclusively, resources could be wasted controlling insignificant or non-existent risks. If grouping criteria are set too restrictively, chemicals exerting joint toxicity might be excluded and risks could be overlooked. To protect against possible mixture-related effects in regulatory assessment, some have called for the application of a “mixtures allocation factor” [33]. The value of the mixtures allocation factor is mixture-specific, can be defined by exposure data, and is set to ensure a level of protection that is similar to what is currently used in safety assessments for individual chemicals. As more scientists incorporate robust mixture study designs into their research, whether in traditional models of toxicity or in NAM-based approaches, uncertainty in mixture assessment will be better characterized and eventually reduced. The articles contained herein support a number of key themes, including (1) mixtures can elicit effects that are different from individual component-induced effects; (2) complex mixtures, which may be more representative of environmental exposures than simple mixtures, can have significant effects beyond those elicited by more simplistic mixtures; (3) more research is needed on mixtures of both chemical and non-chemical stressors to mimic real-world scenarios; (4) evaluating these realistic aspects of mixtures and accurately predicting complex mixture effects continues to present regulatory challenges; and (5) the application of emerging methodologies will be paramount to future mixture assessment and management. While these aspects make the study of mixtures incredibly complex, it is clear from these studies that mixture assessments are crucial to accurately estimating health risks.

## References

[1] Rajapakse N., Silva E., Kortenkamp A. (2002). Combining xenoestrogens at levels below individual no-observed-effect concentrations dramatically enhances steroid hormone action. Environ. Health Perspect..

[2] Silva E., Rajapakse N., Kortenkamp A. (2002). Something from “nothing”—Eight weak estrogenic chemicals combined at concentrations below NOECs produce significant mixture effects. Environ. Sci. Technol..

[3] Martin O., Scholze M., Ermler S., McPhie J., Bopp S., Kienzler A., Parissis N., Kortenkamp A. (2021). Ten years of research on synergisms and antagonisms in chemical mixtures: A systematic review and quantitative reappraisal of mixture studies. Environ. Int..

[4] Wang A., Abrahamsson D.P., Jiang T., Wang M., Morello-Frosch R., Park J.S., Sirota M., Woodruff T.J. (2021). Suspect Screening, Prioritization, and Confirmation of Environmental Chemicals in Maternal-Newborn Pairs from San Francisco. Environ. Sci. Technol..

[5] Park Y.H., Lee K., Soltow Q.A., Strobel F.H., Brigham K.L., Parker R.E., Wilson M.E., Sutliff R.L., Mansfield K.G., Wachtman L.M. (2012). High-performance metabolic profiling of plasma from seven mammalian species for simultaneous environmental chemical surveillance and bioeffect monitoring. Toxicology.

[6] Soltow Q.A., Strobel F.H., Mansfield K.G., Wachtman L., Park Y., Jones D.P. (2013). High-performance metabolic profiling with dual chromatography-Fourier-transform mass spectrometry (DC-FTMS) for study of the exposome. Metabolomics.

[7] Frediani J.K., Jones D.P., Tukvadze N., Uppal K., Sanikidze E., Kipiani M., Tran V.T., Hebbar G., Walker D.I., Kempker R.R. (2014). Plasma metabolomics in human pulmonary tuberculosis disease: A pilot study. PLoS ONE.

[8] Wang Z., Walker G.W., Muir D.C.G., Nagatani-Yoshida K. (2020). Toward a Global Understanding of Chemical Pollution: A First Comprehensive Analysis of National and Regional Chemical Inventories. Environ. Sci. Technol..

[9] Yim G., Wang Y., Howe C.G., Romano M.E. (2022). Exposure to Metal Mixtures in Association with Cardiovascular Risk Factors and Outcomes: A Scoping Review. Toxics.

[10] Trevisan R., Ranasinghe P., Jayasundara N., Di Giulio R.T. (2022). Nanoplastics in Aquatic Environments: Impacts on Aquatic Species and Interactions with Environmental Factors and Pollutants. Toxics.

[11] Fletcher E.J., Santacruz-Marquez R., Mourikes V.E., Neff A.M., Laws M.J., Flaws J.A. (2022). Effects of Phthalate Mixtures on Ovarian Folliculogenesis and Steroidogenesis. Toxics.

[12] Zhou C., Gao L., Flaws J.A. (2017). Prenatal exposure to an environmentally relevant phthalate mixture disrupts reproduction in F1 female mice. Toxicol. Appl. Pharmacol..

[13] Scarano W.R., Bedrat A., Alonso-Costa L.G., Aquino A.M., Fantinatti B., Justulin L.A., Barbisan L.F., Freire P.P., Flaws J.A., Bernardo L. (2019). Exposure to an environmentally relevant phthalate mixture during prostate development induces microRNA upregulation and transcriptome modulation in rats. Toxicol. Sci. Off. J. Soc. Toxicol..

[14] Hoffman K., Hammel S.C., Phillips A.L., Lorenzo A.M., Chen A., Calafat A.M., Ye X., Webster T.F., Stapleton H.M. (2018). Biomarkers of exposure to SVOCs in children and their demographic associations: The TESIE Study. Environ. Int..

[15] Drakvik E., Altenburger R., Aoki Y., Backhaus T., Bahadori T., Barouki R., Brack W., Cronin M.T.D., Demeneix B., Hougaard Bennekou S. (2020). Statement on advancing the assessment of chemical mixtures and their risks for human health and the environment. Environ. Int..

[16] Reina-Perez I., Olivas-Martinez A., Mustieles V., Salamanca-Fernandez E., Molina-Molina J.M., Olea N., Fernandez M.F. (2022). The Mixture of Bisphenol-A and Its Substitutes Bisphenol-S and Bisphenol-F Exerts Obesogenic Activity on Human Adipose-Derived Stem Cells. Toxics.

[17] Carberry C.K., Turla T., Koval L.E., Hartwell H., Fry R.C., Rager J.E. (2022). Chemical Mixtures in Household Environments: In Silico Predictions and In Vitro Testing of Potential Joint Action on PPARgamma in Human Liver Cells. Toxics.

[18] Ha K., Xia P., Crump D., Saini A., Harner T., O’Brien J. (2021). Cytotoxic and Transcriptomic Effects in Avian Hepatocytes Exposed to a Complex Mixture from Air Samples, and Their Relation to the Organic Flame Retardant Signature. Toxics.

[19] Merrill A.K., Anderson T., Conrad K., Marvin E., James-Todd T., Cory-Slechta D.A., Sobolewski M. (2021). Protracted Impairment of Maternal Metabolic Health in Mouse Dams Following Pregnancy Exposure to a Mixture of Low Dose Endocrine-Disrupting Chemicals, a Pilot Study. Toxics.

[20] Phillips A.L., Hammel S.C., Konstantinov A., Stapleton H.M. (2017). Characterization of Individual Isopropylated and tert-Butylated Triarylphosphate (ITP and TBPP) Isomers in Several Commercial Flame Retardant Mixtures and House Dust Standard Reference Material SRM 2585. Environ. Sci. Technol..

[21] Gillera S.E.A., Marinello W.P., Nelson M.A., Horman B.M., Patisaul H.B. (2022). Individual and Combined Effects of Paternal Deprivation and Developmental Exposure to Firemaster 550 on Socio-Emotional Behavior in Prairie Voles. Toxics.

[22] Gore A.C., Moore T., Groom M.J., Thompson L.M. (2022). Prenatal Exposure to an EDC Mixture, NeuroMix: Effects on Brain, Behavior, and Stress Responsiveness in Rats. Toxics.

[23] Fey M.E., Goodrum P.E., Razavi N.R., Whipps C.M., Fernando S., Anderson J.K. (2022). Is Mixtures’ Additivity Supported by Empirical Data? A Case Study of Developmental Toxicity of PFOS and 6:2 FTS in Wildtype Zebrafish Embryos. Toxics.

[24] Kassotis C.D., LeFauve M.K., Chiang Y.T., Knuth M.M., Schkoda S., Kullman S.W. (2022). Nonylphenol Polyethoxylates Enhance Adipose Deposition in Developmentally Exposed Zebrafish. Toxics.

[25] Ctverackova L., Jancula D., Raska J., Babica P., Sovadinova I. (2020). Structure-Dependent Effects of Phthalates on Intercellular and Intracellular Communication in Liver Oval Cells. Int. J. Mol. Sci..

[26] Gray L.E., Ostby J., Furr J., Price M., Veeramachaneni D.N., Parks L. (2000). Perinatal exposure to the phthalates DEHP, BBP, and DINP, but not DEP, DMP, or DOTP, alters sexual differentiation of the male rat. Toxicol. Sci..

[27] United States Environmental Protection Agency (U.S. EPA) (2000). Supplementary Guidance for Conducting Health Risk Assessment of Chemical Mixtures. https://cfpub.epa.gov/ncea/risk/recordisplay.cfm?deid=20533.

[28] Conley J.M., Lambright C.S., Evans N., Medlock-Kakaley E., Dixon A., Hill D., McCord J., Strynar M.J., Ford J., Gray L.E. (2022). Cumulative maternal and neonatal effects of combined exposure to a mixture of perfluorooctanoic acid (PFOA) and perfluorooctane sulfonic acid (PFOS) during pregnancy in the Sprague-Dawley rat. Environ. Int..

[29] Howdeshell K.L., Rider C.V., Wilson V.S., Furr J.R., Lambright C.R., Gray L.E. (2015). Dose Addition Models Based on Biologically Relevant Reductions in Fetal Testosterone Accurately Predict Postnatal Reproductive Tract Alterations by a Phthalate Mixture in Rats. Toxicol. Sci..

[30] Christiansen S., Scholze M., Dalgaard M., Vinggaard A.M., Axelstad M., Kortenkamp A., Hass U. (2009). Synergistic disruption of external male sex organ development by a mixture of four antiandrogens. Environ. Health Perspect..

[31] Kjaerstad M.B., Taxvig C., Andersen H.R., Nellemann C. (2010). Mixture effects of endocrine disrupting compounds in vitro. Int. J. Androl..

[32] Dinse G.E., Umbach D.M. (2011). Characterizing non-constant relative potency. Regul. Toxicol. Pharmacol..

[33] Backhaus T. The Mixture Assessment or Allocation Factor: Conceptual Background, Estimation Algorithms and a Case Study Example. 2022. Conceptual Background, Estimation Algorithms and a Case Study Example, 2022. https://www.researchsquare.com/article/rs-1986611/v1.

